# Controlling Individuals Growth in Semantic Genetic Programming through Elitist Replacement

**DOI:** 10.1155/2016/8326760

**Published:** 2015-12-27

**Authors:** Mauro Castelli, Leonardo Vanneschi, Aleš Popovič

**Affiliations:** ^1^NOVA IMS, Universidade Nova de Lisboa, 1070-312 Lisboa, Portugal; ^2^Faculty of Economics, University of Ljubljana, Kardeljeva Ploščad 17, 1000 Ljubljana, Slovenia

## Abstract

In 2012, Moraglio and coauthors introduced new genetic operators for Genetic Programming, called geometric semantic genetic operators. They have the very interesting advantage of inducing a unimodal error surface for any supervised learning problem. At the same time, they have the important drawback of generating very large data models that are usually very hard to understand and interpret. The objective of this work is to alleviate this drawback, still maintaining the advantage. More in particular, we propose an elitist version of geometric semantic operators, in which offspring are accepted in the new population only if they have better fitness than their parents. We present experimental evidence, on five complex real-life test problems, that this simple idea allows us to obtain results of a comparable quality (in terms of fitness), but with much smaller data models, compared to the standard geometric semantic operators. In the final part of the paper, we also explain the reason why we consider this a significant improvement, showing that the proposed elitist operators generate manageable models, while the models generated by the standard operators are so large in size that they can be considered unmanageable.

## 1. Introduction

In the original definition of Genetic Programming (GP) [1, 2], the operators used to explore the search space, crossover, and mutation produce offspring by manipulating the syntax of the parents. In the last few years, researchers have dedicated several efforts to the definition of new GP systems based on the semantics of the solutions [3–5]. Differently from other domains [6–8], in the field of GP the term semantics refers to the behavior of a program once it is executed or more particularly the set of its output values on input training data [9–11]. In particular, new genetic operators, called geometric semantic operators, have been proposed by Moraglio and coauthors [9]. While these operators have interesting properties, which make them a very interesting GP hot topic [9, 12], they present an important limitation: at each application, the newly created individuals have a size that is bigger than the one of the parents. Even using implementation that allows executing the system in a very efficient way (like the one presented in [12]), the problem persists, in the sense that it is in general practically impossible to fully reconstruct the final model generated by GP and whenever it is possible, the resulting expression is so big that it cannot be understood by a human being. For this reason, in this study we define a very simple but effective method that allows GP to produce more compact solutions, without affecting the quality of the final solutions. More in detail, we propose to keep the offspring into the new population only in case they have a better fitness than the parents, keeping the parents otherwise. This type of “elitist” strategy, which has already been applied to standard “syntax-based” GP operators, has, to the best of our knowledge, never been applied to geometric semantic operators before.

The paper is organized as follows: Section 2 defines the geometric semantic operators first introduced in [9]; Section 3 first experimentally analyzes some characteristics of these operators and then presents the proposed elitist method.  Section 4 presents the experimental settings and the obtained results, showing the appropriateness of the proposed technique in reducing individuals growth without penalizing their fitness. Finally, Section 5 concludes the paper and provides hints for possible future work.

## 2. Geometric Semantic Operators

Even though the term semantics can have several different interpretations, it is a common trend in the GP community (and this is what we do also here) to identify the semantics of a solution with the vector of its output values on the training data [3, 11, 13]. Under this perspective, a GP individual can be identified with a point (its semantics) in a multidimensional space that we call semantic space. The term Geometric Semantic Genetic Programming (GSGP) indicates a recently introduced variant of GP in which traditional crossover and mutation operators are replaced by so-called geometric semantic operators, which exploit semantic awareness and induce precise geometric properties on the semantic space. Geometric semantic operators, introduced by Moraglio et al. [9], are becoming more and more popular in the GP community [3] because of their property of inducing a unimodal fitness landscape on any problem consisting in matching sets of input data into known targets (like supervised learning problems such as regression and classification). Geometric semantic operators define transformations on the syntax of the individuals that correspond to geometric crossover and ball mutation [11] in the semantic space. Geometric crossover is an operator of Genetic Algorithms (GAs) that generates an offspring that has, as coordinates, weighted averages of the corresponding coordinates of the parents with weights smaller than one, whose sum is equal to one. Ball mutation is a variation operator that slightly perturbs some of the coordinates of a solution. Geometric crossover generates offspring that stand on the segment joining the parents. It is possible to prove that, in all cases where fitness is a monotonic function of a distance to a given target, the offspring of geometric crossover cannot be worse than the worst of its parents, while ball mutation induces a unimodal fitness landscape.

*Geometric semantic crossover* (here we report the definition of the geometric semantic operators as given by Moraglio et al. for real functions domains, since these are the operators we will use in the experimental phase. For applications that consider other types of data, the reader is referred to [9]) generates the expression *T*_*XO*_ = (*T*_1_ · *T*_*R*_)+((1 − *T*_*R*_) · *T*_2_) as the unique offspring of parents *T*_1_, *T*_2_ : *ℝ* ^*n*^ → *ℝ*, where *T*_*R*_ is a random real function whose output values range in the interval [0,1]. Analogously,* geometric semantic mutation* returns the expression *T*_*M*_ = *T* + ms · (*T*_*R*1_ − *T*_*R*2_) as the result of the mutation of an individual *T* : *ℝ* ^*n*^ → *ℝ*, where *T*_*R*1_ and *T*_*R*2_ are random real functions with codomain in [0,1] and ms is a parameter called mutation step.

As Moraglio et al. point out, these operators create much larger offspring than their parents and the fast growth of the individuals in the population rapidly makes fitness evaluation unbearably slow, making the system unusable. In [12], a possible workaround to this problem was proposed consisting in implementation of Moraglio's operators that makes them not only usable in practice, but also very efficient. Basically, this implementation is based on the idea that, besides storing the initial trees, at every generation it is enough to maintain in memory, for each individual, its semantics and a reference to its parents. As shown in [12], the computational cost of evolving a population of *n* individuals for *g* generations is *O*(*ng*), while the cost of evaluating a new, unseen, instance is *O*(*g*). This allows GP practitioners to use geometric semantic operators to address complex real-life problems [14].

Geometric semantic operators have a known limitation [3]: the reconstruction of the best individual at the end of a run can be a hard (and sometimes even impossible) task, due to its large size.

## 3. Elitist Geometric Semantic Operators

### 3.1. Issues with Geometric Semantic Operators and Motivations

Even though in [9] Moraglio and coworkers presented interesting results on a set of benchmarks, clearly showing that geometric semantic operators are very promising, they also pointed out an important drawback of these operators: when these operators are used, the size of the individuals in the population grows very rapidly. In order to give an intuitive idea of the importance of the phenomenon, let us consider two individuals *T*_1_ and *T*_2_ and let us assume that these individuals belong to the initial population of a GP run. By the definition of geometric semantic crossover, the offspring of the crossover between *T*_1_ and *T*_2_ is

(1) $$\begin{array}{c} XO\left(\;T_{1},T_{2}\;\right)=\left(\;T_{1}*T_{R}\;\right)+\left(\;\left(\;1-T_{R}\;\right)*T_{2}\;\right), \end{array}$$

where *T*_*R*_ is a random tree. Assuming for simplicity that GP uses only crossover (the reasoning can be easily extended to the case of mutation), at generation 2 all the individuals in the population will have a shape like the one of the individual in (1), with the only difference that different trees will be plugged in place of *T*_1_, *T*_2_, and *T*_*R*_.

If we iterate this reasoning, performing the crossover between two individuals belonging to the population at generation 2, the offspring has the following shape:

(2) XOT1∗TR1+1−TR1∗T2,T3∗TR2+1−TR2∗T4=T1∗TR1+1−TR1∗T2∗TR3+1−TR3∗T3∗TR2+1−TR2∗T4

and all the individuals at generation 3 will share this structure, although using different trees instead of *T*_1_, *T*_2_, *T*_3_, *T*_4_, and *T*_*R*1_, *T*_*R*2_. While individuals of the same shape as the ones in (2) may already seem complex and hard to read, let us now assume that we iterate the GP run for hundreds of generations. It is not difficult to understand that the individuals in the population rapidly become so large that they are completely unreadable. Furthermore, evaluating those individuals at each generation would make the GP run extremely slow.

Moraglio and coauthors have an interesting discussion about code growth in [9], and they show that in the case of geometric semantic crossover this growth has even an exponential speed. This clearly represents a problem for the usability of GP and the readability of the generated individuals. Solving, or at least alleviating, this problem is the motivation of the present work.

### 3.2. Principle of Elitist Geometric Semantic Operators

To partially counteract this problem we propose the following replacement method:

(i) Considering two parents *P*_1_ and *P*_2_, the offspring *O*_cross_ obtained from the semantic crossover between *P*_1_ and *P*_2_ is accepted in the new population if and only if its fitness is better than the fitness of both *P*_1_ and *P*_2_. Otherwise, one of the two parents is copied in the new population. As we will make clear in the continuation, the parent that survives can be a random one or the best among *P*_1_ and *P*_2_, and the choice between these two options has a very weak effect on the overall performance of the system.
(ii) Considering an individual *T*, the offspring *O*_mut_ obtained applying semantic mutation on *T* is accepted in the new population if and only if its fitness is better than the fitness of *T*. In the opposite case, *T* itself is copied in the new population.

While the idea is quite simple, it is interesting to point out that the proposed method is supposed to be useful if the geometric semantic genetic operators tend to produce a high number of individuals whose fitness is not better than the fitness of their parents. Hence, before testing the proposed method, it makes sense to perform an experimental analysis aimed at understanding how many crossover and mutation events produce an offspring with a better fitness than the parents. In order to do that, we considered five real-life applications: three applications in the field of drug discovery that are becoming widely used benchmarks for GP [15], an application related to the prediction of the high performance concrete strength [16], and a medical application whose objective is predicting the seriousness of the symptoms of a set of Parkinson disease patients, based on an analysis of their voice [17]. Regarding the three drug discovery applications, the objective is to predict three important pharmacokinetic parameters of molecular compounds that are candidate to become new drugs: human oral bioavailability (%F), median lethal dose (LD50), and protein plasma binding level (PPB). These problems have already been tackled by GP in published literature and for a discussion of them the reader is referred to [10]. Regarding the size of the dataset, the %F dataset consists of a matrix of 260 rows (instances) and 242 columns (features). The LD50 consists of 234 instances and 627 features, while the PPB dataset consists of 131 instances and 627 features. Each row is a vector of molecular descriptor values identifying a drug; each column represents a molecular descriptor, except the last one, which contains the known target values of the considered pharmacokinetic parameter. Regarding the concrete dataset, it consists of 9 features and 1030 instances. Each row is a vector of concrete-related characteristics. The Parkinson dataset consists of 19 features and ≈6000 instances.

For this experimental study, we use the same experimental settings considered in [12]. The only difference (besides the number of generations) is that, in this study, we considered several mutation step values. This is fundamental considering that we want to analyze the behavior of the semantic mutation operator whose performance is influenced by the mutation step value.

**Figure 1:**
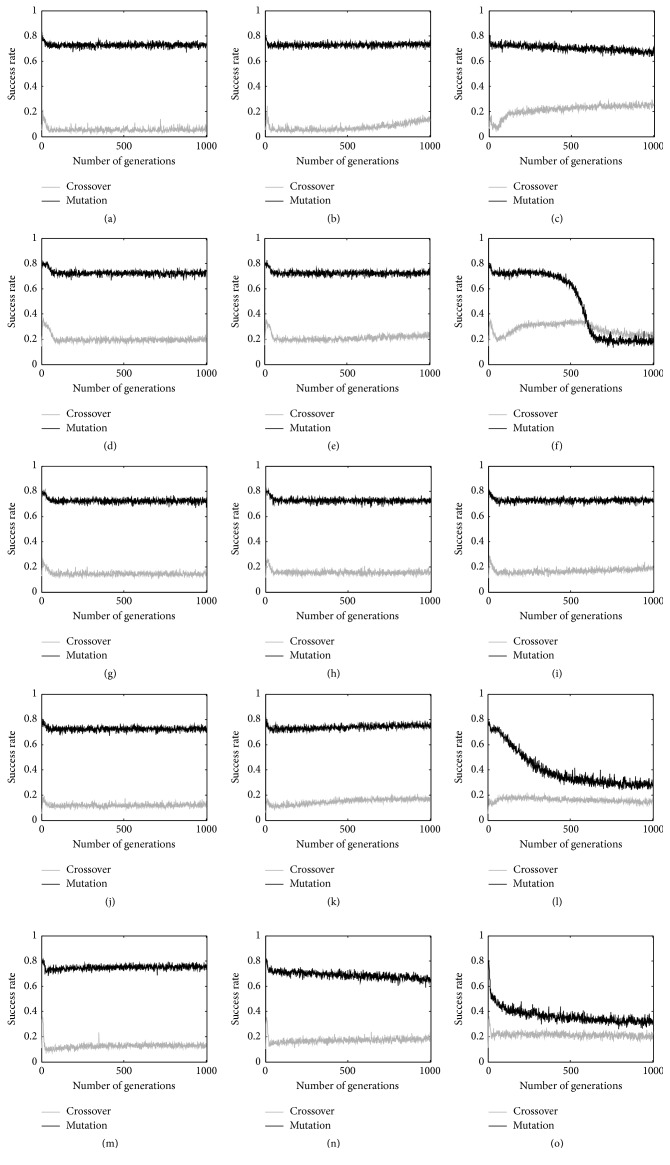
Success rate of the crossover and mutation events. For each one of the five considered problems (from top to the bottom: %F, PPB, LD50, concrete, and Parkinson), three different mutation steps have been considered. From left to right, ms = 0.01, ms = 0.1, and ms = 1.

Results of this analysis are reported in Figure 1. In particular, we reported, for all the considered problems, the median (calculated over 30 independent runs) of the percentage of crossover and mutation events that have produced an offspring with a fitness better than the respective parents. Let us discuss these results problem by problem, and, for each problem, considering the different mutation steps. For the %F problem with ms = 0.01 (Figure 1(a)) and ms = 0.1 (Figure 1(b)), we can draw similar observations: the mutation operator produces an offspring that has a better fitness with respect to the original individual in a percentage of the mutation events that stands between 70% and 75%. Regarding the crossover operator, the percentage of successful crossover events stands between 8% and 15%, according to the particular generation that is considered. A slightly different behavior can be observed in Figure 1(c), where a mutation step equal to 1 has been considered. In this case, the crossover operator succeeds in producing offspring with a better fitness than both parents, on average 20% of the times, and it is possible to see an increase of this percentage during the evolution. On the other hand, the percentage of successful mutations decreases, but still mutation succeeds in producing better offspring, on average 70% of the times. A similar analysis can be performed for the second studied problem: PPB. With ms = 0.01 (Figure 1(d)) and ms = 0.1 (Figure 1(e)), crossover is successful on 20% of the events, while mutation produces better offspring (with respect to their parents) in 75% of the cases. When a mutation step equal to 1 is considered (Figure 1(f)), the mutation success rate decreases during the evolution, passing from effectiveness of the 80% in the initial generations to a final 20%. Also, the success rate of crossover changes along the evolutionary process, starting with 40% and then passing to 30% and with a final rate of 20%. The LD50 problem presents a similar behavior for all the considered mutation step values (from Figures 1(g)–1(i)): mutation produces fitter individuals in 75% of the applications and crossover in approximately 20%. Finally, the concrete problem and the Parkinson problem present a similar behavior between each other, and thus they can be discussed together. For ms = 0.01 and ms = 0.1 crossover produces better individuals than both the parents in a percentage between 10% and 25% of the applications, while mutation produces fitter individuals in a percentage between 65% and 80% of the cases. Similar conclusions can be drawn when a mutation step equal to 1 is considered. The only difference in this last case is that the percentage of mutation events that produce fitter individuals rapidly decreases, passing from the initial 80% to the final 30%. On the other hand, crossover produces a better individual than both parents in only 20% of the cases.

It is worth pointing out that this last situation is ideal for testing the effectiveness of the proposed growth control method. In fact, the proposed technique will be applied in 80% of the crossover events, hence reducing by a significant amount the size of the individuals in the population. At the same time, considering semantic mutation, it is possible to notice that the mutation events produced a better offspring than the parent in the large majority of the cases. Hence, the proposed technique will be less effective with mutation. However, it will still contribute to reducing the average size of the individuals in the population. Moreover, it is known that the semantic crossover operator is mainly responsible for the growth of the size of the individuals; hence it makes sense that a method aimed at controlling the size of the individuals is applied principally to crossover.

## 4. Experimental Study

In this section, we analyze the training and test performance of GSGP with the proposed elitist replacement technique, comparing it to standard GSGP. The results of this study are reported in Section 4.1. Then, we present a study aimed at understanding the actual contribution, in terms of size reduction, given by the proposed elitist replacement method. The latter study is presented in Section 4.2. To perform all the experiments, we used the implementation of the geometric semantic operators available at http://gsgp.sourceforge.net/.

### 4.1. Training and Test Fitness

The plots shown in this section report as fitness the root mean square error between target and obtained values on training and test data. All the results have been obtained by considering 30 independent runs and the plots report the median fitness of the best individual (on the training set) at each generation. Each run uses a different partition of the data: 70% of the instances, selected at random with uniform distribution, form the training set, while the remaining 30% are used to assess the performance of the model on unseen instances. The obtained results will be discussed separately for each studied test problem, and, for each one of them, the results obtained using different values of the mutation step will be analyzed.

**Figure 2:**
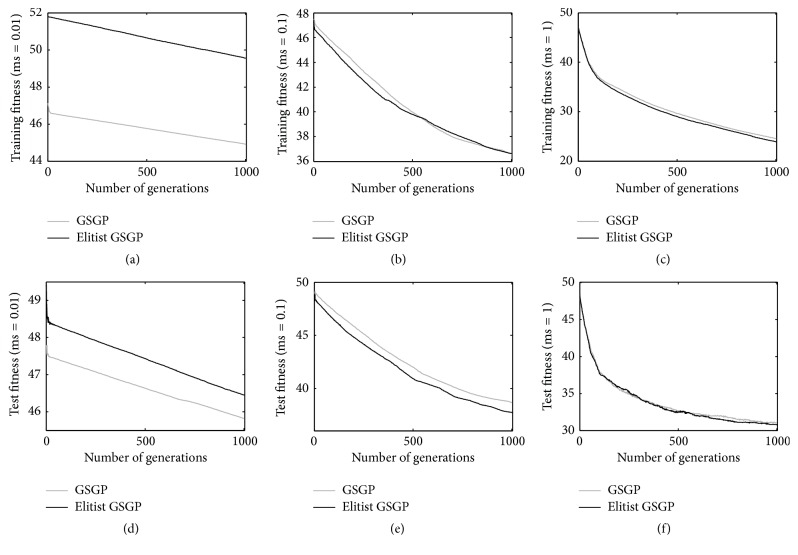
Training (plots a, b, and c) and test (plots d, e, and f) fitness for the %F problem, considering different mutation step values.

Figure 2 shows the results achieved for the %F dataset. Considering a mutation step of 0.01 (Figures 2(a) and 2(d)), it is possible to see that GSGP produces better performance on both the training and test instances with respect to the proposed elitist replacement technique. However, the situation is different when the remaining mutation steps are considered: with a mutation step of 0.1 (Figures 2(b) and 2(e)) the two techniques produce results of comparable quality on the training set, but the elitist method outperforms GSGP on the test set. Finally, the two considered GP systems return comparable results when a mutation step equal to 1 is considered (Figures 2(c) and 2(f)). This is particularly important, considering that as reported in the literature [12], for the %F problem the best results are achieved considering a mutation step equal to 1. In other words, on the %F problem, the proposed method is able to perform as well as GSGP on both training and test data, while maintaining in the population smaller individuals (as it will be clear in the continuation).

**Figure 3:**
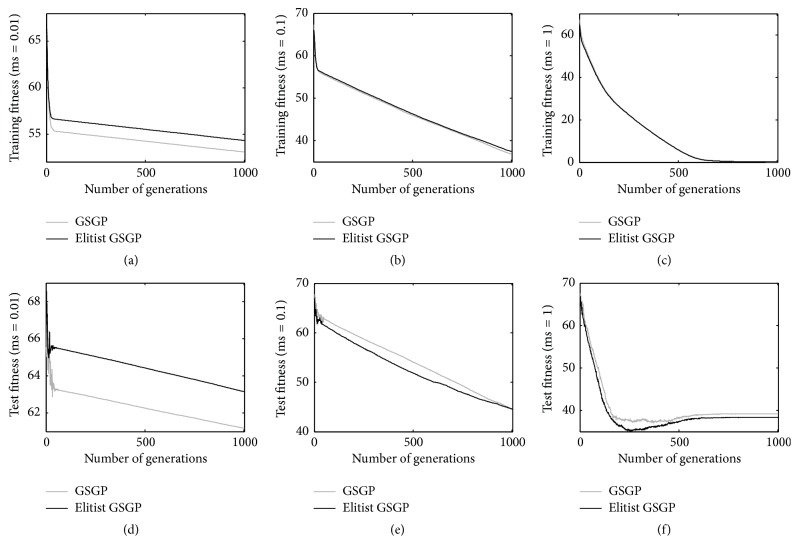
Training (plots a, b, and c) and test (plots d, e, and f) fitness for the PPB problem considering different mutation step values.

The same observations can be drawn considering the PPB dataset (results reported in Figure 3). Also in this case, GSGP outperforms the elitist replacement technique on both the training and test sets when a mutation step equal to 0.01 is considered (Figures 3(a) and 3(d)). On the other hand, with a mutation step equal to 0.1 (Figures 3(b) and 3(e)) and with a mutation step equal to 1 (Figures 2(c) and 2(f)), the considered GP systems produce comparable results on both training and test instances. Also for this problem, it is important to highlight that the best performances are achieved with a mutation step equal to 1 [12] and, in this case, the elitist replacement method is able to perform as well as GSGP on both training and test data.

**Figure 4:**
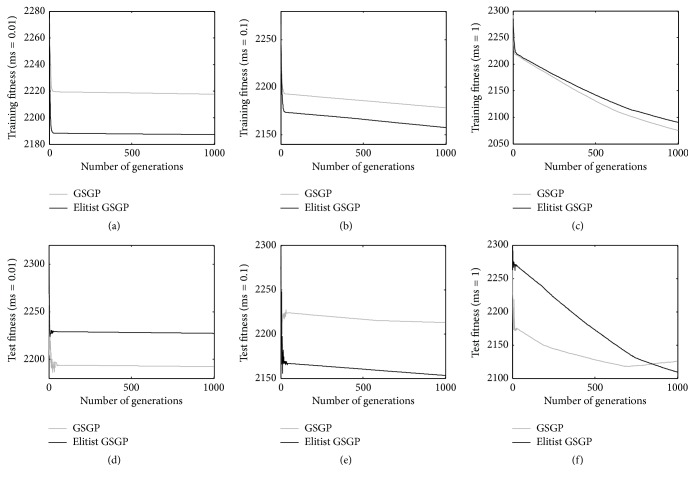
Training (plots a, b, and c) and test (plots d, e, and f) fitness for the LD50 problem considering different mutation step values.

For the third considered problem, that is, the LD50 dataset (results reported in Figure 4), we observe a different behavior. In this case, with a mutation step of 0.01 (Figures 4(a) and 4(d)), the elitist method outperforms GSGP on the training set, but, on the other hand, it is outperformed by GSGP on the test set. With a mutation step equal to 0.1 (Figures 4(b) and 4(e)), the proposed elitist replacement method outperforms GSGP on both the training and test instances. Anyway, also for the case of the LD50 dataset, the most interesting results are the ones achieved with the mutation step equal to 1, which is the value that has allowed us to find the best results so far for this problem [12]. In this case, the two considered GP systems produce comparable results on the training instances (Figure 4(c)). Interestingly, as Figure 4(f) shows, their behaviors on the unseen instances are very different between each other: GSGP is able to reach better fitness values faster than the elitist method. On the other hand, considering the last few studied generations, GSGP is not able to further improve the results, while the elitist technique seems to have some further room for improvement. In other words, at the end of the run both methods return comparable results on the test set, but the elitist technique obtains those results later in the run.

**Figure 5:**
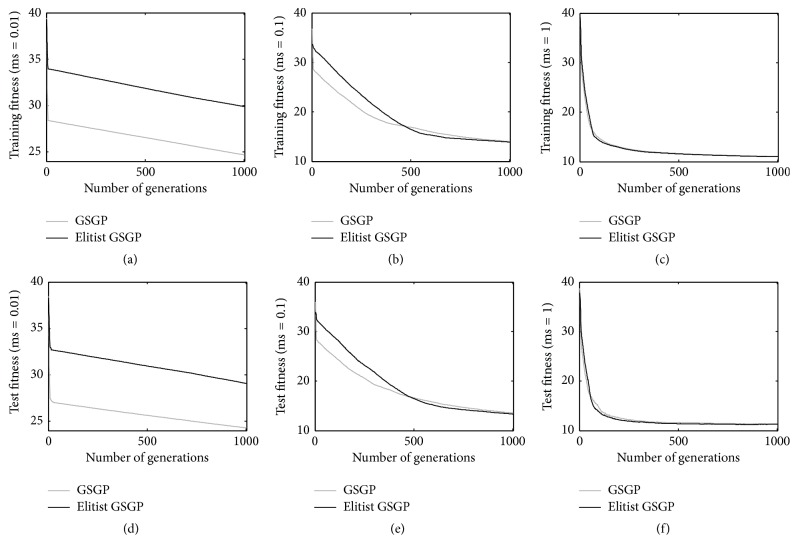
Training (plots a, b, and c) and test (plots d, e, and f) fitness for the concrete problem considering different mutation step values.

For the concrete dataset (results shown in Figure 5), it is possible to draw conclusions that are quite similar to the ones reported for the %F problem. In particular, considering a mutation step of 0.01 (Figures 5(a) and 5(d)), it is possible to see that GSGP obtains better results on both the training and test sets. On the other hand, with a mutation step equal to 0.1 (Figures 5(b) and 5(e)) and with a mutation step equal to 1 (Figures 5(c) and 5(f)), the considered GP systems produce results that are comparable, on both the training and test sets. Also for this problem, it is important to note that the best performance is achieved using a mutation step equal to 1 and, in this case, the elitist replacement method is able to perform as well as GSGP on both the training and test instances.

**Figure 6:**
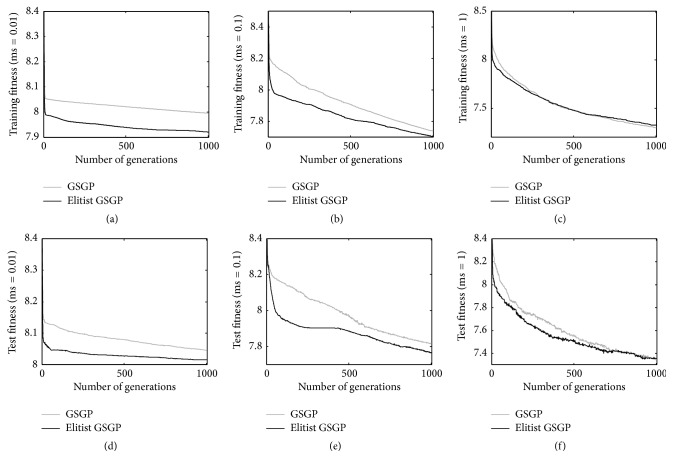
Training (plots a, b, and c) and test (plots d, e, and f) fitness for the Parkinson problem considering different mutation step values.

Figure 6 shows the results achieved on the Parkinson dataset. Considering a mutation step of 0.01 (Figures 6(a) and 6(d)) and a mutation step of 0.1 (Figures 6(b) and 6(e)), we observe that the proposed elitist replacement technique outperforms GSGP on both the training and test sets. Nevertheless, when a mutation step equal to 1 is considered (Figures 6(c) and 6(f)), the two systems produce comparable results, on both the training and test data. Also in this case, this is an important aspect, considering that, also for the Parkinson dataset, the best results known so far have been achieved considering a mutation step value equal to 1 (as reported in [10]). Hence, also in this last application, the proposed elitist method is able to perform as well as GSGP on both training and test data when the best known value of the mutation step is used.

To summarize all of these results, we point out that, for all the studied applications, the two GP systems perform differently (in some cases GSGP outperforms the elitist method and in other cases vice versa) when small values (i.e., 0.01 and 0.1) of the mutation step are considered. However, the two systems always produce comparable results when a mutation step equal to 1 is considered. This value of the mutation step is known from the literature to be the one that allows GSGP to produce the best results known so far for all the considered problems. Hence, when the best setting for the mutation step is used, the elitist method always produces results that are comparable to the ones obtained using GSGP.

**Figure 7:**
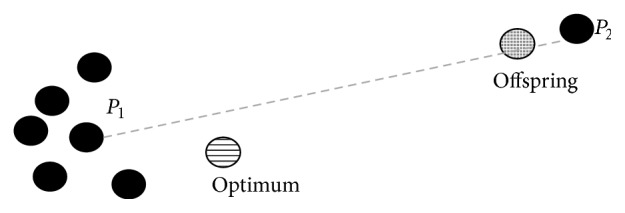
Example of crossover that generates an offspring whose fitness is not better than the fitness of both the parents.

The differences between GSGP and the proposed elitist method, observed when mutation steps equal to 0.01 and 0.1 have been considered, can be explained with the following argument: when a small mutation step is considered, the mutation operator creates individuals with semantics that differ from the original parents by very small quantities (the variation quantity is limited by the value of ms itself). Hence, in this case, crossover is the operator that produces the strongest effect on the search process (i.e., crossover has a larger exploration power). In this scenario, individuals created by crossover can give an important contribution in approaching the global optimum even if their fitness is not better than the fitness of both parents. The situation can be sketched in Figure 7. Even though this is an extremely simplified example (e.g., the semantic space is bidimensional here, while, having the same dimension as the number of fitness cases, it is highly multidimensional in the studied test cases), this figure helps to give an idea of the fact that, with the elitist method, it would be difficult to reach the optimum by using only the crossover operator. In fact, the elitist method will ignore all the individuals like the offspring reported in the figure. On the other hand, with the standard GSGP system, it is possible to generate an offspring like the one represented in the figure. Hence, using an informal argument, we could say that crossover can create several individuals that are “on the right side” of the figure, hence incrementing the probability of selecting, in the next generation of the search process, two individuals containing the optimum in the segment joining them.

### 4.2. Average Individuals Size

**Figure 8:**
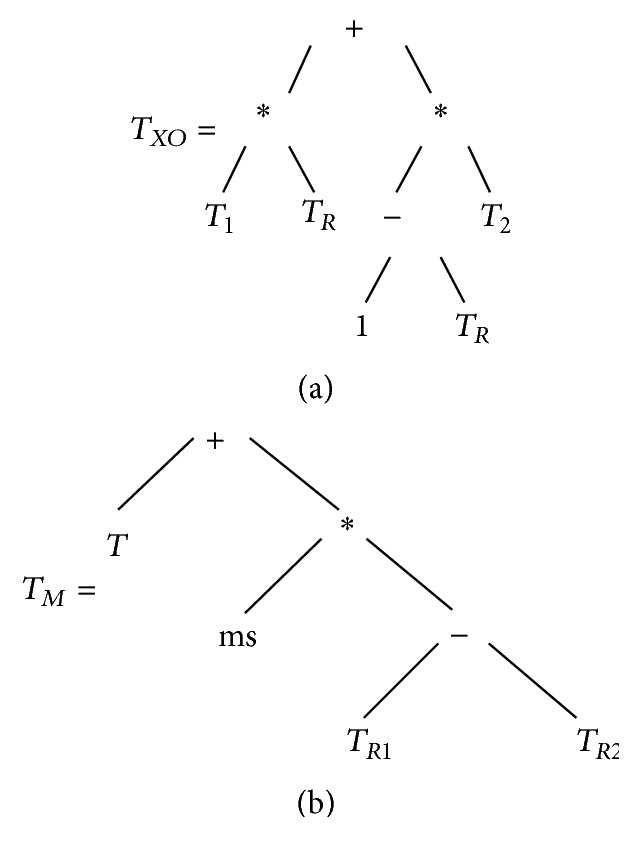
Individuals generated by the geometric semantic crossover (a) and by the geometric semantic mutation (b).

While it is computationally expensive to calculate the exact size of each tree in the population, it is possible to perform a theoretical study that, with a good approximation, allows us to gain some information about the average size of the individuals at a certain generation. Let us consider again the definition of geometric semantic crossover and mutation. In particular let us consider the structure of the individuals that are created by the semantic operators. Starting from 2 parents *T*_1_ and *T*_2_ the geometric semantic crossover produces an offspring *T*_*XO*_ that has the structure shown in Figure 8(a). The offspring consists of a copy of the genotype of the parent individuals, plus 5 nodes and two copies of the genotype of one random tree *T*_*R*_, whose maximum depth is known (let us denote it as *D*). Analogously, starting from an individual *T*, the geometric semantic mutation produces an offspring *T*_*M*_ that has the structure shown in Figure 8(b). It is composed of a copy of the genotype of *T*, plus the genotype of 2 different random trees (whose maximum depth is, again, *D*) and 4 nodes. Hence, denoting as *p*_*xo*_ the crossover probability, with *p*_*m*_ the mutation probability and with *p*_*r*_ the reproduction probability (where *p*_*xo*_ + *p*_*m*_ + *p*_*r*_ = 1), the average size of the individuals after *g* generations (indicated as $\hat{S}(g)$) has an upper bound (given by the fact that we pessimistically consider the depth of all the used random trees equal to *D*, which is instead the maximum possible value); that is, this formula holds only if all the primitive functions used by GP are binary operators (which allows us to state that the number of nodes of a tree of depth *D* is 2^*D*^). In the experiments presented in this paper, the used primitive functions were always the binary arithmetic operators, so this property is respected:

(3) S^g=pxo·2·S^g−1+2·2D+5+pm·S^g−1+2·2D+4+pr·S^g−1.

From this formula, which is problem independent, it is clear that as expected, reproduction gives a smaller contribution to size of the individuals than crossover and mutation. Given that the proposed elitist method, contrary to GSGP, performs reproduction instead of crossover approximately 85% of the times and reproduction instead of mutation approximately 25% of the times, it is clear that it maintains in the population smaller individuals.

**Figure 9:**
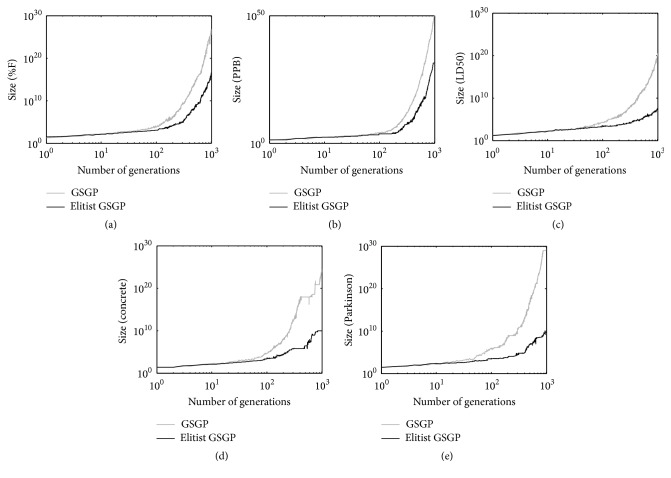
Evolution of the median size of the best individuals in the population calculated over 30 independent runs.

In order to give experimental corroboration to this finding, in Figure 9 we report the evolution of the median size of the best individual in the population (calculated over 30 independent runs), while in Table 1 we report the median size of the best individuals at termination (final models) for the five test problems considered previously. The proposed elitist method actually maintains populations of individuals that are smaller than the ones produced by the standard GSGP system. To assess the statistical significance of the difference between the size of the individuals produced by the proposed elitist method and the ones produced by the standard GSGP system, we performed a set of statistical tests on the median size. As a first step, the Shapiro Wilk test (with *α* = 0.1) has shown that the data are not normally distributed and hence a rank-based statistic has been used. Then, the Mann-Whitney test has been used. The null hypotheses for the comparison across repeated measures are that the distributions (whatever they are) are the same across repeated measures. The alternative hypotheses are that distributions across repeated measures are different. Also in this test a value of *α* = 0.1 has been used. The *p* values returned by the statistical test have shown that the elitist method produced individuals whose size is significantly smaller than the size of the individuals obtained with the standard GSGP system.

Once established, both theoretically and experimentally, that the proposed elitist method maintains populations of smaller individuals than GSGP, it is interesting to discuss if the size of those individuals is “usable” or as it typically happens for GSGP, the individuals are still too big to be managed. To answer this question, we use the following argument: in his first book on GP, Koza established a fixed tree depth limit of the individuals in the population equal to 17. Even though this limit appears quite arbitrary, several GP studies even nowadays use this limit that has become, more or less, standard value for the maximum tree depth. As such, we can state that a population that contains individuals with a depth equal to 17 is still “usable” population. If we use only binary primitives, like in this work, trees of depth equal to 17 have a number of nodes equal to 2^17^. Both from (3) and from the curves of Figure 9, we can see that GSGP begins to have in the population individuals with a number of nodes approximately equal to 2^17^ around generation 100, while for the proposed elitist method this happens around generation 500. From the literature [9, 12], we know that, despite the fact that GSGP induces a unimodal fitness landscape, it is able to navigate it using very small optimization steps. Also, the experimental results reported in the literature in which GSGP is compared to standard GP for the problems studied here [12] indicate that 100 generations are not enough for GSGP to outperform standard GP, while 500 generations are.

In conclusion, the proposed elitist method outperforms standard GP and obtains results that are qualitatively comparable to the ones of GSGP, but, contrary to what happens for GSGP, it is able to maintain populations composed of individuals of manageable size.

## 5. Conclusions

Recent work in Genetic Programming (GP) has been dedicated to the definition of methods based on the semantics of the solutions. Among the existing semantic-based methods, one of the most recent methods is based on the definition of particular genetic operators, called geometric semantic genetic operators, that have precise consequences on the semantics of the individuals. This GP variant, known as Geometric Semantic GP (GSGP), has shown very interesting results for a vast set of complex real-life applications in several domains, consistently outperforming standard GP on all of them. Nevertheless, an important problem affects GSGP: the geometric semantic operators, by construction, generate individuals that are larger than their parents, leading rapidly to unmanageable populations unless very specific implementation is used. Also, the very big dimension of the individuals makes it difficult to read and understand the final solution, practically turning GP into a black box system. To limit this important drawback of GSGP, in this paper we proposed a method (called elitist system) in which a newly created individual is accepted as a member of the new population only if it has a fitness that is better than the fitness of the parents. A preliminary experimental analysis, presented in the first part of the paper, has shown that in GSGP several applications of the genetic operators do not produce offspring that are better than their parents (in particular for crossover). This fact has encouraged us to pursue the research and implement the proposed elitist system. The experimental results that we have presented in the central part of the paper have shown that the proposed elitist system produces individuals of comparable quality to the ones obtained with standard GSGP. The final part of the paper was dedicated to a comparison between the size of the individuals maintained in the population by the proposed elitist method and the ones of GSGP. This study, besides confirming, as it was expected, that the proposed elitist method creates smaller individuals, has also indicated that the individuals evolved by the elitist method maintain a manageable size at least until generation 500, while for GSGP this is true only approximately until generation 100. In this study, we have used as a threshold between manageable and unmanageable individuals sizes the depth limit equal to 17 suggested by Koza in his first book on GP. Given that as known from the literature, geometric semantic operators allow us to outperform standard GP only in the late stages of the evolution, we can conclude that when GSGP outperforms standard GP, its populations are already unmanageable, while the proposed elitist method is able to outperform standard GP while still evolving individuals of manageable size. This is the real advantage of the proposed elitist method compared to GSGP: for comparable levels of performance, individuals are not simply “smaller” but “significantly smaller” in the sense that the difference in size fills the gap between the possibilities of storing the individuals in memory and using them or not.

A lot of future work is planned on this research track, with the final objective of defining a GSGP system able to maintain the same geometric properties of the current one, but in which individuals do not steadily grow during the evolution. If, on the one hand, it is important to further test the elitist method proposed in this paper on several other applications, possibly in conjunction with several other improvements, on the other hand simplification methods aimed at maintaining optimized expressions in the population deserve investigation. Extending the achievements obtained so far by GSGP on symbolic regression to other kinds of application is also a priority. Geometric semantic genetic operators for different applicative domains, like Boolean problems and classification, were already defined in the original work of Moraglio and coauthors and have been later further refined by the same authors. We are currently working toward the definition of geometric semantic operators for applications in the field of pattern reconstruction, like the artificial ant on the Santa Fe trail. Preliminary experimental results seem to indicate that the proposed elitist method may be particularly useful for these new operators. Last but not least, the most ambitious task of this research track remains to be the definition of new geometric semantic operators that, while maintaining the same geometric properties of the current ones, do not create individuals that are larger than their parents. An important first step has already been taken by Moraglio and collaborators, with the definition of such operators for the particular domain of basis functions. An extension of this result to functions of any possible shape is one of the main objectives of our current research.

## Figures and Tables

**Table 1:** Size of the best model after 1000 generations. Median calculated over 30 runs.

	Size	
	GSGP	Elitist GSGP
%F	6.65*E* + 26	3.80*E* + 16
PPB	8.37*E* + 49	2.39*E* + 31
LD50	9.59*E* + 19	2.28*E* + 07
Concrete	5.36*E* + 23	9.43*E* + 09
Parkinson	1.10*E* + 29	4.47*E* + 09
